# Phospholipid: diacylglycerol acyltransferase contributes to the conversion of membrane lipids into triacylglycerol in *Myrmecia incisa* during the nitrogen starvation stress

**DOI:** 10.1038/srep26610

**Published:** 2016-05-24

**Authors:** Xiao-Yu Liu, Long-Ling Ouyang, Zhi-Gang Zhou

**Affiliations:** 1 College of Aqua-life Sciences and Technology, Shanghai Ocean University, Shanghai 201306, China

## Abstract

In addition to the Kennedy pathway for *de novo* biosynthesis, triacylglycerol (TAG), the most important stock for microalgae-based biodiesel production, can be synthesized by phospholipid: diacylglycerol acyltransferase (PDAT) that transfers an acyl group from phospholipids (PLs) to diacylglycerol (DAG). This study presents a novel gene that encodes PDAT from the green microalga *Myrmecia incisa* Reisigl H4301 (designated *MiPDAT* ). MiPDAT is localized on the plasma membrane (PM) via the agroinfiltration of tobacco leaves with a green fluorescent protein-fused construct. MiPDAT synthesizes TAG based on functional complementary experiments in the mutant yeast strain H1246 and the membrane lipid phosphatidylcholine (PC) is preferentially used as substrates as revealed by *in vitro* enzyme activity assay. The gradually increased transcription levels of *MiPDAT* in *M*. *incisa* during the cultivation under nitrogen starvation conditions is proposed to be responsible for the decrease and increase of the PC and TAG levels, respectively, as detected by liquid chromatography-mass spectrometry after 4 d of nitrogen starvation. In addition, the mechanism by which MiPDAT in this microalga uses PC to yield TAG is discussed. Accordingly, it is concluded that this PM-located PDAT contributes to the conversion of membrane lipids into TAG in *M*. *incisa* during the nitrogen starvation stress.

Microalgae are a promising source of nutrients and renewable biofuels because of their storage lipids^1^,^2^,^3^. The primary storage lipid is triacylglycerol (TAG), which is synthesized via multiple pathways in eukaryotes. The *de novo* biosynthesis of TAG by the Kennedy pathway involves the sequential acylation of the glycerol backbone with acyl-CoA via three acyltransferases, glycerol-3-phosphate acyltransferase (GPAT, EC 2.3.1.15), lysophosphatidic acid acyltransferase (LPAAT, EC 2.3.1.51), and acyltransferase: diacylglycerol acyltransferase (DGAT, EC 2.3.1.20)^4^,^5^,^6^,^7^,^8^. In addition, there is another pathway that synthesizes TAG catalysed by phospholipid: diacylglycerol acyltransferase (PDAT, EC 2.3.1.158). This enzyme transfers an acyl group from the *sn*-2 position of phospholipids (PLs) to the *sn*-3 position of diacylglycerol (DAG), yielding *sn*-1-lysophospholipid and TAG, respectively. This pathway has been documented in yeast^9^, higher plants^10^,^11^, and the microalga *Chlamydomonas reinhardtii*^12^. In microalgae, particularly those grown under nitrogen deficiency conditions, the accumulation of TAG is often accompanied by membrane lipid degradation^2^,^13^,^14^,^15^. Accordingly, PDAT may be involved in lipid trafficking in *C*. *reinhardtii*^12^.

Similar to *C*. *reinhardtii*, *Myrmecia incisa*^16^, an arachidonic acid-rich green microalga^17^, forms an abundance of oil bodies after cultivation under nitrogen starvation conditions^18^. Each oil body has a core of non-polar lipid TAG surrounded by a monolayer of amphipathic phospholipids and structural proteins^19^,^20^,^21^,^22^, indicating that TAG is the major component of oil bodies. Recently, Chen *et al*.^23^ revealed that the isoform DGAT2A was responsible for the increase of TAG by *de novo* synthesis with acyl-CoA. Because of the sparse thylakoid membrane and reduced size of chloroplasts in *M*. *incisa* during the nitrogen starvation stress^18^, we determined whether the enzyme PDAT contributes to TAG accumulation.

In this study, a full-length complementary DNA (cDNA) encoding PDAT (designated *MiPDAT*) was cloned from *M*. *incisa*, and its function was identified by a complementary experiment in the TAG-deficient strain H1246 of *Saccharomyces cerevisiae*. It was shown that MiPDAT used phosphatidylcholine (PC) to synthesize TAG indirectly by feeding the transgenic yeast with various fatty acids (FAs) as substrates and directly by *in vitro* enzyme activity assay. The *in vivo* evidence for the function of this gene in *M*. *incisa* was provided by estimating the variation of PLs and *MiPDAT* transcription levels using liquid chromatography-mass spectrometry (LC-MS) and quantitative real-time PCR (Q-RT-PCR), respectively, in this microalga during the nitrogen starvation stress. Furthermore, to determine the subcellular localization of MiPDAT, this gene was fused with the green fluorescent protein (*GFP*) gene to construct the vector p1300-MiPDAT-GFP. This vector was then infiltrated into the tobacco leaf mediated by *Agrobacterium tumefaciens* GV3101. It is concluded from these data that MiPDAT contributes to TAG accumulation by converting membrane PC in *M*. *incisa* grown under nitrogen starvation stress.

## Results

### Cloning and characterization of a PDAT gene from *M*. *incisa*

**Figure 1:**
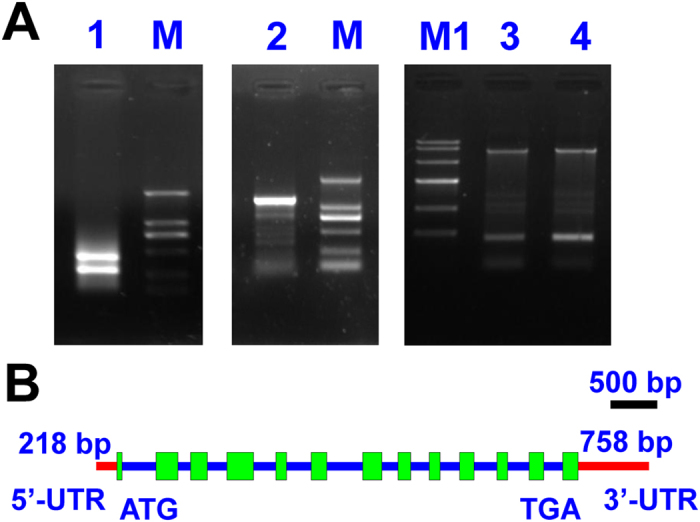
Agarose gel electrophoretogram and gene structure of *MiPDAT*. (**A**) Agarose gel electrophoretogram of PCR products generated from the full-length cDNA and DNA cloning of *MiPDAT*. M: DL 2000 DNA standard marker; M1: DNA Marker IV; Lane 1: PCR products of 5′-RACE; Lane 2: PCR products of 3′-RACE; Lanes 3 and 4: PCR products of DNA cloning. (**B**) Schematic illustration of the gene structure of *MiPDAT*. The green boxes represent exons. A total of 12 introns with lengths of 361 bp, 136 bp, 215 bp, 239 bp, 266 bp, 384 bp, 176 bp, 199 bp, 211 bp, 246 bp, 227 bp and 209 bp are presented as the blue line. Red lines represent the un-translated region (UTR).

Seven contigs, contigs 1820, 4542, 7311, 7384, 8397, 13344, and 15617, from the transcriptome database of *M*. *incisa*^24^ were determined to be homologous to the gene *PDAT*. From the manual assembly of these contigs, a 2,939-bp fragment was obtained and verified by PCR amplification. Based on this fragment sequence, two pairs of primers (GSP5-1 and NGSP5-1, GSP3-1 and NGSP3-1, Supplementary Table S1) were designed, and then, a 1,162-bp 3′-rapid-amplification of cDNA ends (RACE) product and a 467-bp 5′-RACE product (Fig. 1A) were amplified and sequenced. These amplified products shared overlapping regions at their corresponding ends of the 2,939-bp assembled fragment. After manual assembly of these sequences, a 3,079-bp full-length cDNA consisting of a 2,076-bp open reading frame (ORF), 218-bp 5′-untranslated region (UTR), and 785-bp 3′-UTR was obtained and designated *MiPDAT*. The ORF of *MiPDAT* was predicted to encode a protein consisting of 691 amino acids with a molecular weight of 77.27 kD. This putative protein MiPDAT had 50%, 32%, and 34% similarities with the PDATs from *Chlamydomonas reinhardtii* (GenBank accession No. AFB73928), *Saccharomyces cerevisiae* (GenBank accession No. DAA10549), and *Arabidopsis thaliana* (GenBank accession No. AED91921), respectively.

To characterize the gene structure of *MiPDAT*, the corresponding genomic DNA of *MiPDAT* was amplified with one pair of designed primers (MiPDAT-OS and MiPDAT-OA, Supplementary Table S1). After cloning and comparison of the corresponding cDNA and DNA sequences, it was revealed that *MiPDAT* was separated by 12 introns with lengths of 361 bp, 136 bp, 215 bp, 239 bp, 266 bp, 384 bp, 176 bp, 199 bp, 211 bp, 246 bp, 227 bp, and 209 bp from the 5′-terminus (Fig. 1B). All of the introns had splicing consensus GT-AG borders. Both the cDNA and DNA sequences of *MiPDAT* were deposited in GenBank under accession Nos KU851950 and KU871014, respectively.

### Homologous alignment and phylogenetic inference of MiPDAT

By searching InterPro, a lecithin: cholesterol acyltransferase (LCAT, EC 2.3.1.43) domain (Phe128-Asp690) in the putative MiPDAT was identified, suggesting that it belonged to the LCAT family (Pfam: 02450). Therefore, two LCAT and ten other PDAT homologs were included in the multiple sequence alignment (Supplementary Fig. S1), showing at least 7 reserved domains.

Of these domains, Domain II, designated the lid domain by Peelman *et al*.^25^, was closed by a disulphide bridge, as detected between the two nearly neighbouring residues Cys171 and Cys196 in MiPDAT, corresponding to Cys74 and Cys98 in human LCAT. This bridge was highly conserved and covered a hydrophobic active site in all of the PDATs. The residue Trp in this lid domain was also conserved in MiPDAT and others (Supplementary Fig. S1), and it was predicted to bind cleaved FAs in the active site of these enzymes^26^.

The highly conserved Domain III, which contains a salt bridge between Asp258 and Arg260, may be involved in PL recognition^12^. A catalytic triad of Ser205, Asp369, and His401 distributed in Domains IV, VI, and VII, respectively, of MiPDAT was also conserved in both the LCATs and PDATs. This triad is a part of the catalytic domain of the LCAT enzymes, in which a FA is transesterified from PC to cholesterol to yield a cholesterol ester^12^,^25^. There was a consensus sequence, GHSXG, which is part of a conserved lipase motif, in Domain IV of all LCATs^27^. The first Gly in the domain of these LCATs was, however, different from that in the plant PDATs, and it was replaced by Ser in algal PDATs or Pro in higher plants (Supplementary Fig. S1). Compared to the domains discussed above, the roles of the relatively conserved Domains I and V are poorly understood in PDATs.

The neighbour-joining phylogenetic tree (Supplementary Fig. S2) inferred from MiPDAT and other LCAT-like family proteins supported the conclusion^12^,^28^ that the LCAT-like family proteins from higher plants, animals, fungi, and algae could be divided into four major groups. This phylogenetic tree (Supplementary Fig. S2) also showed that MiPDAT was grouped into PDATs, which was supported by a bootstrap value of 99%, and that it was closer to the microalgal PDATs, including a function-identified *Chlamydomonas* PDAT^12^, than the higher plant PDATs.

Based upon these characteristics and the phylogenetic analysis, it was suggested that the cloned gene *MiPDAT* from *M*. *incisa* should function as a PDAT to yield TAG by transferring an acyl group from PLs.

### Functional expression of *MiPDAT* in *Saccharomyces cerevisiae* H1246

**Figure 2:**
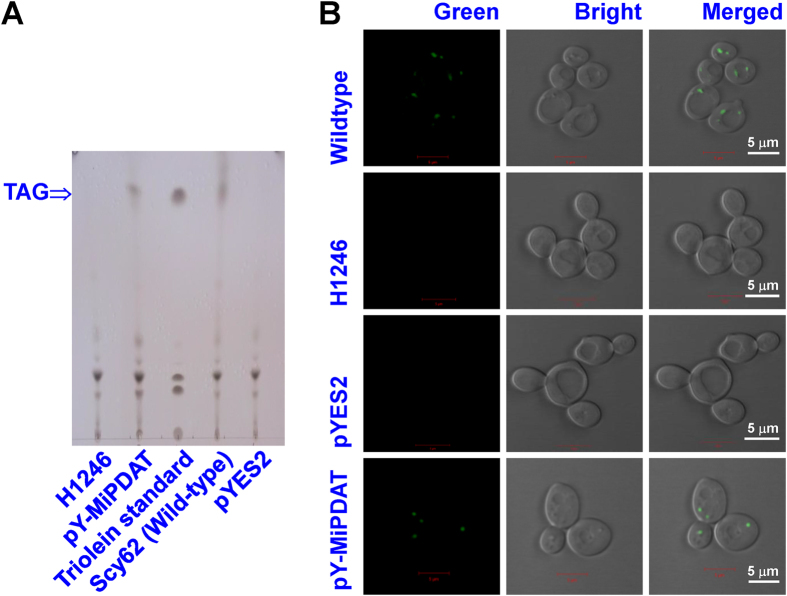
Functional identification of *MiPDAT* in *Saccharomyces cerevisiae* H1246. (**A**) Lipid analysis of *Saccharomyces cerevisiae* by TLC. Lane 1: H1246 mutant; Lane 2: H1246 mutant transformed with pY-MiPDAT; Lane 3: triolein standard purchased from Nu Chek Prep, Inc. (UK); Lane 4: SCY62 (wild-type); Lane 5: H1246 transformed with empty pYES2. (**B**) Fluorescent staining of yeast cells with BODIPY. Lipid bodies where neutral lipids accumulated were visualized in the yeast cells with BODIPY fluorescence. The wild-type strain Scy62 was used as a positive control. The mutant H1246 and the mutant harbouring the empty vector (pYES2) were used as negative controls. The mutant expressing MiPDAT was analysed. All bars in the image B represent a length of 5 μm.

To identify the function of the protein encoded by *MiPDAT*, the ORF of this gene was used to generate a recombinant plasmid, pY-MiPDAT (Supplementary Fig. S3), for complementary experiments in the TAG-deficient mutant yeast strain H1246^29^. TAG, in transgenic or wild-type lines, was detected by thin layer chromatography (TLC) analysis, and oil bodies stained with BODIPY were observed. As shown in the TLC profile (Fig. 2A), a prominent spot corresponding to a TAG standard occurred in the recombinant line with *MiPDAT* comparable to the wild type, whereas there was no TAG formed in the mutant strain H1246 or the negative control only carrying empty vector. This result demonstrated that the TAG-deficient mutant recovered TAG synthesis after *MiPDAT* was introduced, indicating that MiPDAT can synthesize TAG.

TAG is usually present in the form of oil bodies in both yeast and plants^19^,^20^,^21^,^22^. Therefore, observations on the formation of oil bodies in yeast may be beneficial for the understanding of MiPDAT function. Using the unique staining agent BODIPY^23^, oil bodies were observed in wild-type Scy62 (the original strain of H1246 29) cells and the recombinant line with *MiPDAT*, but they were absent in the negative control and mutant strain H1246 (Fig. 2B). Apparently, this introduced gene led to the formation oil bodies in the mutant yeast cells, confirming the TAG synthesis function of MiPDAT, similar to the recently reported DGATs in *M*. *incisa*^23^.

### Substrate preference analysis of MiPDAT

**Figure 3:**
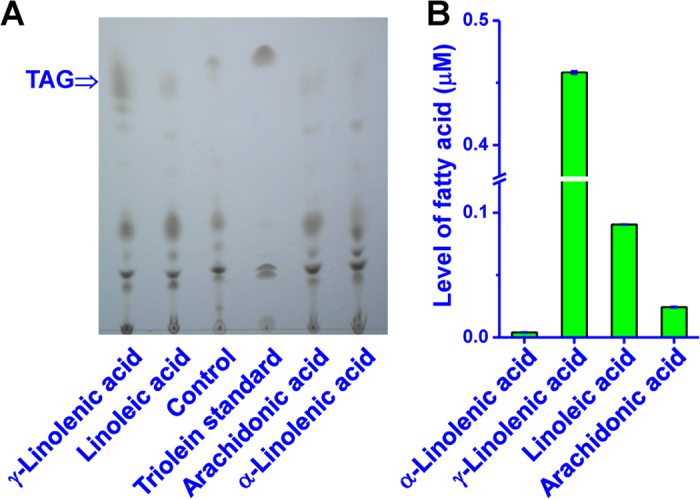
Substrate preference analysis of MiPDAT. (**A**) Lipid analysis of the substrate preference in *Saccharomyces cerevisiae* with pY-MiPDAT by TLC. Lane 1: recombinant line inoculated in medium with GLA; Lane 2: recombinant line inoculated in medium with LA; Lane 3: recombinant line inoculated in medium with no exogenous fatty acids; Lane 4: triolein standard purchased from Nu Chek Prep, Inc. (UK); Lane 5: recombinant line inoculated in medium with ArA; Lane 6: introduce medium with ALA. (**B**) Fatty acid levels in the isolated TAG as developed in the image A. Each bar represents the mean ± SD for triplicate experiments.

To better understand whether MiPDAT has a preference for FAs for the synthesis of TAG, the recombinant yeast carrying pY-MiPDAT was fed various FAs selected primarily based on their presence in *M*. *incisa*^17^. These exogenous FAs were separately added as substrates to the SC-uracil induction medium for yeast cultivation. TAG extracted from the recombinant line carrying pY-MiPDAT was detected by TLC analysis (Fig. 3A). The intensity of the coloured spots corresponding to the TAG (Fig. 3A) differed in each sample cultivated with different substrates, suggesting that this recombinant line had a substrate preference. Quantitative analysis by gas chromatography-mass spectrometry (GC-MS) indicated that this recombinant line used γ-linolenic acid (GLA) preferentially to synthesize TAG because the level of GLA in the TAG (Fig. 3B) from the same weight of lyophilized yeasts was significantly (*P* < 0.01) higher than that of other FAs. Additionally, MiPDAT had a significant (*P* < 0.01) preference for linoleic acid (LA), arachidonic acid (ArA), and α-linolenic acid (ALA) (Fig. 3B). This finding was consistent with the preference analysis of *Linum usitatissimum* PDAT^11^.

**Figure 4:**
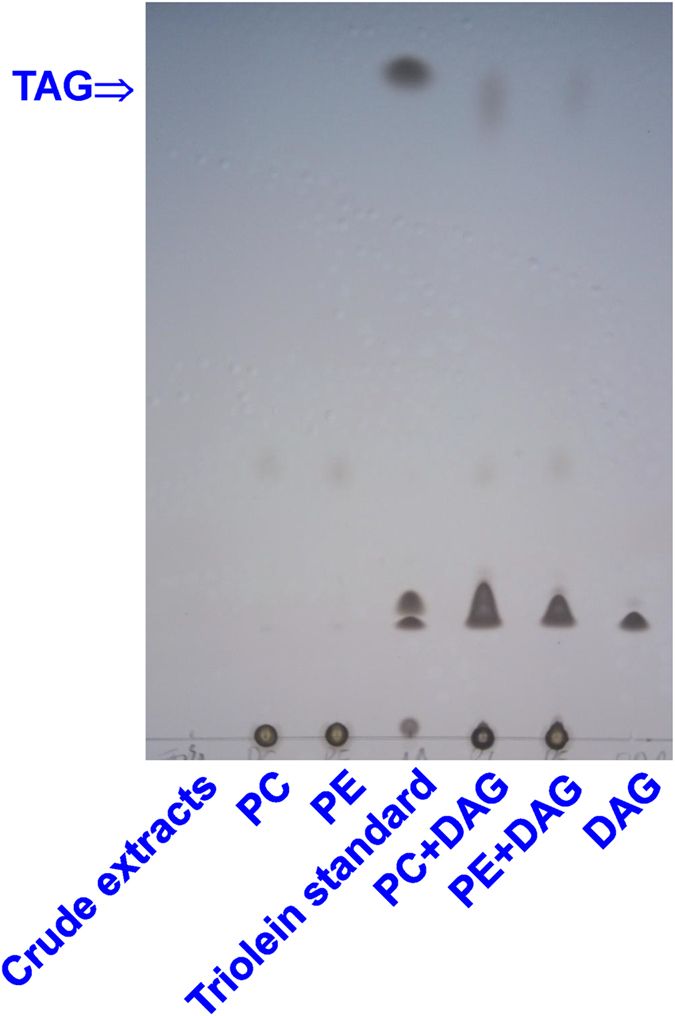
*In vitro* enzyme activity assay for MiPDAT. The diacylglycerol (DAG) with phosphatidylcholine (PC) or phosphatidylethanolamine (PE) was used as substrates. The crude extracts alone and the lipids donors or acceptor DAG with the crude extracts were used as negative controls. Triolein standard purchased from Nu Chek Prep, Inc. (UK).

*In vitro* enzyme activity assays could directly reveal the preference for PLs used by MiPDAT in TAG synthesis. As shown in Fig. 4, there was one prominent spot corresponding to the TAG standard occurred when PC+DAG or phosphatidylethanolamine (PE)+DAG were mixed and incubated with transgenic yeast crude extracts. The abundance of TAG synthesized from PC seemed to be more than that from PE (Fig. 4). By contrast, there was no formed TAG while either one of two lipid donors or acceptor was mixed with the crude extracts. Neither was there while incubating with the crude extracts alone (Fig. 4). This result indicated that MiPDAT could use PC and PE to synthesize TAG, and MiPDAT had a preference for PC over PE.

### Effect of nitrogen starvation on the content of microalgal phospholipids

PLs usually decrease in microalgae, particularly when grown under nitrogen starvation^2^,^13^,^14^,^15^. A culture during the nitrogen starvation stress was therefore performed to investigate the variation of PLs as detected by LC-MS in *M*. *incisa*. A principle component analysis (PCA) model with two components was constructed and showed that the lipid samples designated 4 d group (culture under nitrogen starvation for 4 d) were clearly separated from those designated 0 d group (culture at the onset of nitrogen starvation) (Supplementary Fig. S4A). In addition, the discriminant analysis, orthogonal projection to latent structures with discriminant analysis (OPLS-DA) [R^2^Y(cum) = 0.991 and Q^2^(cum) = 0.948] model with high R^2^Y(cum) and Q^2^(cum) values also provided reliable support for the separation of the 0 d group from the 4 d group (Supplementary Fig. S4B). It was concluded that *M*. *incisa* varied significantly in its lipid profile after cultivation under nitrogen starvation for 4 d.

To identify the lipid that contributed to the separation of the nitrogen starvation cultivation group for 4 d from the 0 d group, 84 potential lipid biomarkers [variable importance in the projection (VIP) ranging from 3.57 to 1.00] were selected according to the VIP values as well as the corresponding 95% confidence intervals based on a jack-knife procedure and the absolute value of p(corr)^30^,^31^. Of these biomarkers, the plasma-membrane lipids, PE and PC, accounted for 54.76% of the total (46 to 84) and possessed relatively high VIPs (ranging from 1.01 to 3.40), indicating that these two PLs were the main contributors to this separation.

**Figure 5:**
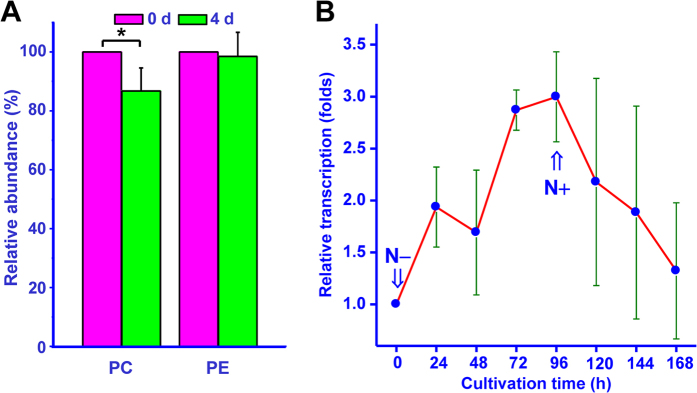
Variations of the phospholipid (PL) levels and the transcriptional levels of *MiPDAT* in *M*. *incisa* during nitrogen starvation stress. (**A**) Comparison of the total relative abundance of all phosphatidylcholine (PC) and phosphatidylethanolamine (PE) species in *M*. *incisa* grown under nitrogen starvation stress for 0 d or 4 d. Each bar represents the mean ± SD for seven experiments. The asterisk above the column denotes very significantly different (*P* < 0.01) from the 0 d culture at the onset of nitrogen starvation. (**B**) The transcriptional level of *MiPDAT* in *M*. *incisa* during the shift from nitrogen starvation to replenishment conditions. The arrows indicate the onset of nitrogen starvation or replenishment.

Furthermore, the PC and PE contents were compared between the 0 d and 4 d groups of *M*. *incisa* cultured under nitrogen starvation stress. As shown in Fig. 5A, the PC level decreased by 13% in the microalga after 4 day nitrogen starvation cultivation and the PE level decreased by only 1%. These results suggest that PC rather than PE should be converted to other lipids when *M*. *incisa* is cultured during the nitrogen starvation stress.

### The transcription of *MiPDAT* in *M*. *incisa* grown under nitrogen starvation and replenishment conditions

If MiPDAT is related to TAG accumulation and/or PLs degradation in *M*. *incisa* grown during the shift from nitrogen starvation to rich in BG-11 medium, the transcription of this gene should vary correspondingly. To determine this variation, the gene transcription of *MiPDAT* was detected using Q-RT-PCR. As shown in Fig. 5B, with nitrogen starvation, the abundance of transcripts for *MiPDAT* in *M*. *incisa* was increased with the duration of this culture condition and the level increased until reaching a maximal level at 96 h, when it was approximately 3-fold higher than at 0 h (*P* < 0.01). After the onset of nitrogen replenishment, the transcription of this gene decreased gradually, and the level of transcripts at 168 h was only 43% of the level at 96 h (*P* < 0.01). This result indicated that *MiPDAT* was up- and down-regulated in *M*. *incisa* by nitrogen starvation and nitrogen replenishment, respectively, and this regular alternation showed the same trend as the TAG level as reported by Chen *et al*.^23^, suggesting that the transcription of *MiPDAT* is positively related to TAG accumulation.

### Subcellular localization of MiPDAT in tobacco

MiPDAT may link TAG accumulation with PL degradation in *M*. *incisa* during the course of nitrogen starvation; therefore, it was hypothesized that MiPDAT is localized to the membrane to easily use PLs. The subcellular localization of MiPDAT was predicted to determine this. A transmembrane domain (Phe70-Ala92) was predicted by TMHMM analysis, suggesting that MiPDAT is a membrane-bound protein. Neither signal peptide nor transit peptide was predicted by the SignalP 4.1 Server, TargetP 1.1 Server, or the ChloroP 1.1 Server, indicating that this PDAT is not a plastid-localized PDAT, which is different from the PDAT in *C*. *reinhardtii*^12^.

**Figure 6:**
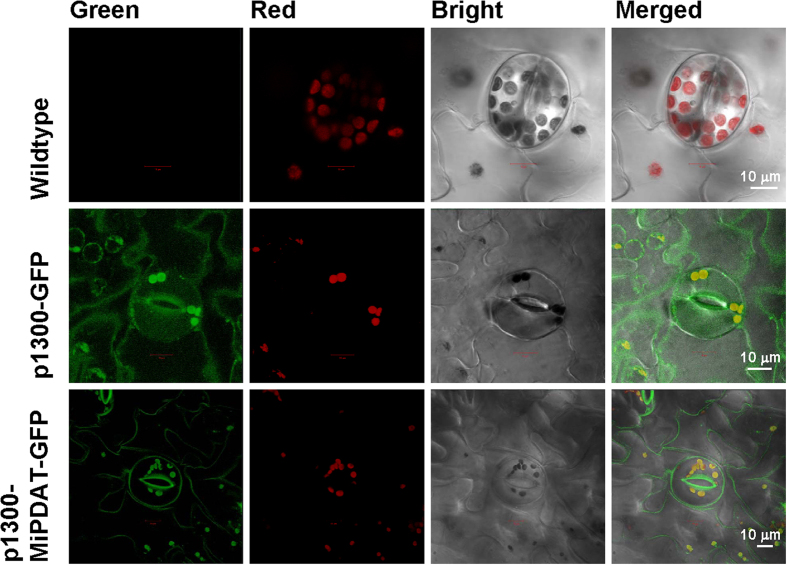
Subcellular localization of MiPDAT in tobacco leaves detected with a confocal laser scanning microscope. A sequence encoding GFP was fused downstream to the *MiPDAT* coding region, which possessed no stop codon. Wild-type tobacco leaves were used as negative controls. The fluorescent image of chloroplast autofluorescence is shown in red, whereas the fluorescent image of GFP is shown in green.

To clarify the subcellular localization of MiPDAT, the complete ORF of this gene was fused with *GFP* to generate a binary vector (Supplementary Fig. S5) and then infiltrated into the lower epidermal cells of tobacco leaves via *Agrobacterium tumefaciens* GV3101. The green fluorescence from the GFP was dispersed thoroughly in the protoplasts of the infected cells (Fig. 6). When the construct p1300-GFP was used as a control, the fluorescent signal on the plasma membrane (PM) of these infected cells, as seen in the two guard cells shown in the middle row of Fig. 6, was not distinct from the cytoplasm. By contrast, after *MiPDAT* was introduced, the green fluorescent signal was mainly visualized on the PM of infected cells so that the PM was clearly distinguished from the cytoplasm (Fig. 6). These results indicate that this PDAT from *M*. *incisa* is located on the PM of infected tobacco leaf epidermal cells.

## Discussion

This study presents a novel microalgal *PDAT* cloned from *M*. *incisa* and different from the reported *C*. *reinhardtii* PDAT (designated *CrPDAT*) because the MiPDAT is located on the PM (Fig. 6). The CrPDAT, however, is proposed to be located on chloroplasts based on the presence of a chloroplast transit peptide^12^, although there is no direct evidence for its subcellular localization. Except for this difference, MiPDAT is similar to CrPDAT and others because they possess seven characteristic conserved domains (Supplementary Fig. S1), and this protein is grouped into the PDAT clade, including the function-identified CrPDAT, which is supported by a 99% bootstrap value in the reconstructed phylogenetic inference (Supplementary Fig. S2). Furthermore, the ability of MiPDAT to synthesize TAG is shown by complementary experiments in yeast (Fig. 2). These data indicate that MiPDAT is a member of the PDAT family. In this case, which PL is used by MiPDAT to synthesize TAG?

PDAT transfers an acyl group from the *sn*-2 position of PLs, such as PC, to the *sn*-3 position of diacylglycerol (DAG) to yield TAG^9^,^10^,^12^,^32^. By feeding yeast exogenous FAs for the functional identification of PDAT in *Linum usitatissimum* (designated LuPDAT), Pan *et al*.^11^ found that these added FAs were transformed to lyso-PC by acyl-CoA: lysophosphatidylcholine acyltransferase (LPCAT EC 2.3.1.23)^33^ to yield FAs-PC. As a result, LuPDAT can use the FA-PCs to synthesize TAG in yeast^11^. A similar feeding experiment was performed for this cloned *MiPDAT*, and the preference for GLA (Fig. 3) by the introduced *MiPDAT* was determined, which is similar to that described by Pan *et al*.^11^ Therefore, MiPDAT may use the same FA-PCs as LuPDAT to synthesize TAG. *In vitro* activity assay of the enzyme extracted from the transgenic yeast with *MiPDAT* (Fig. 4) confirms this deduction evidently and MiPDAT has a preference for PC to PE for TAG synthesis. In this case, the level of PC in *M*. *incisa* may be reduced, whereas the level of TAG increases^23^ during the nitrogen starvation culture. This hypothesis is verified by a lipidomics analysis showing that PC is a main contributor to the significantly changed PLs and that the level of PCs significantly decreases (Fig. 5A) in *M*. *incisa* grown under nitrogen starvation stress for 4 d while comparing to that at the onset of nitrogen starvation. Consistent with the changed levels of PC (Fig. 5A) and TAG (Fig. 4 in the ref. 23), the transcription of *MiPDAT* is up- and down- regulated (Fig. 5B) by the onset of nitrogen starvation and replenishment, respectively. Taken together, this study provides temporal evidence that MiPDAT is involved in PC degradation and TAG accumulation in this microalga grown under nitrogen starvation, although the *MiPDAT*-knockout or -knockdown *M*. *incisa* remains to be completed.

In this study, we have observed that MiPDAT is primarily located on the PM of tobacco leaf epidermis cells (Fig. 6) by co-expression with GFP. As discussed above, MiPDAT uses PC to synthesize TAG. There may be a bridge that connects the PM with the endoplasmic reticulum (ER) because the ER is the principal site for PC biosynthesis and TAG accumulation^34^,^35^. The ER is an interconnected network in eukaryotic cells, including microalgae, and membrane contact sites (MCSs) are present between the ER and other organelles^35^,^36^,^37^. The MCSs display a wide variety of functions including, but not limited to, lipid exchange between two compartments^36^. The ER-PM MCSs, therefore, are proposed to provide a possible mechanism by which the PM-located MiPDAT uses the ER-synthesized PC to accumulate TAG in the ER to form oil bodies in *M*. *incisa*. From the transcriptome database of *M*. *incisa*^24^, 4 unigenes (Supplementary Data 1) and 2 unigenes (Supplementary Data 2) coding for ORP and Sec14, respectively, have been searched. These two proteins function on the ER-PM MCSs to extract one lipid molecule from a membrane and deliver it to another^36^. Accordingly, this suggests the possible existence of ER-PM MCSs in *M*. *incisa* although direct evidence has not yet been obtained. This spatial distribution of MCSs suggests that MiPDAT can use membrane PC to synthesize TAG in *M*. *incisa* grown under nitrogen starvation stress.

In addition, the GFP fluorescent signal has seemingly been detected on the transgenic tobacco leaf chloroplasts, although it cannot be distinguished clearly from the negative control (Fig. 6). For the absence of a transit peptide in MiPDAT as predicted above, and because PC is one component of the outer envelope of chloroplasts^38^, MiPDAT may also be located on this outer envelope. In this case, MiPDAT can synthesize TAG by consuming the chloroplast PC, possibly at the ER-chloroplast MCSs^34^,^35^. Two contigs (Supplementary Data 3) coding for coatomer proteins that act on the ER-chloroplast MCSs^34^,^37^ have also been found in the transcriptome database of *M*. *incisa*^24^. However, the chloroplast localization of MiPDAT is not certain, unless new direct evidence using, for example, gold immunoelectron microscopy, is provided.

The temporal and spatial evidence for MiPDAT contributing to the conversion of membrane lipids into TAG in *M*. *incisa* during nitrogen starvation stress is provided. There are many ways that microalgae utilize membrane lipids to synthesize TAG. For example, PC can also be degraded by phospholipases, and the resulting acyl group can be sequentially used to synthesize TAG along the Kennedy pathway^33^. Additional studies are necessary to further understand the physiological roles of PDAT in microalgal cells grown under stress conditions. Because the ER is the general location of TAG accumulation, this PM-located MiPDAT is more significant than the chloroplast-located one for microalgae-based biodiesel production.

## Methods

### *Myrmecia incisa* and culture conditions

The microalga *Myrmecia incisa* Reisigl H4301^16^ was commercially provided by the Culture Collection Algae of Charles University of Prague (CAPU). This alga was cultivated, washed, and harvested as described previously^24^. For the Q-RT-PCR assay, *M*. *incisa* was cultivated, washed, and harvested as described by Yu *et al*.^39^. For lipid analysis, approximately 60 mL of an algal suspension was sampled every 4 d during the cultivation course of nitrogen starvation. The collected microalgal pellets were washed with distilled water, lyophilized, and stored at −80 °C until use.

### Complementary DNA (cDNA) and DNA cloning of *MiPDAT*

Total RNA was extracted from *M*. *incisa* using TRIzol reagent according to the manufacturer’s instructions (Invitrogen, USA). Complementary DNA was synthesized from the total RNA using the PrimeScript™ RT Reagent Kit (TaKaRa, Japan). The full-length cDNA of *MiPDAT* was amplified with a SMART™ RACE cDNA Amplification Kit (Clontech, USA) with two designed pairs primers based on the screened sequences from a high throughput transcriptome of *M*. *incisa*^24^. One pair of primers (GSP5-1 and NGSP5-1) was designed for the 5′-RACE reaction, and the other pair of primers (GSP3-1 and NGSP3-1) was designed for the 3′-RACE reaction (Supplementary Table S1). The amplified products were resolved on a 1.0% low-melting-point agarose gel for DNA recovery. The target product was recovered using agarose gel purification and an extraction kit (Aidlab, China) and was ligated to a pMD19-T vector (TaKaRa, Japan). The constructed vector was subsequently transformed into *Escherichia coli* DH5α competent cells (Tiangen, China), and positive clones were sent to Sangon (China) for sequencing. The DNA was extracted using a modified cetyltrimethylammonium bromide (CTAB) method^40^. Based on the cDNA sequence of *MiPDAT*, genomic DNA of *M*. *incisa* was used as the template for the DNA cloning of this gene with one pair of primers (MiPDAT-OS and MiPDAT-OA, Supplementary Table S1).

### Bioinformatics analysis

The detailed bioinformatics analysis is provided in the Supplementary Methods.

### Heterologous expression of *MiPDAT* in yeast

*Saccharomyces cerevisiae* H1246 (from Prof. Sten Stymne), a quadruple mutant strain lacking four genes, *dga1*, *lro1*, *are1*, and *are2*, for TAG synthesis^29^ was used in the complementary experiments to identify the function of *MiPDAT*. The ORF of *MiPDAT* was cloned with the primers pHindF and pBamR (Supplementary Table S1) designed with *Hin*d III/*Bam*H I digestion sites and then ligated into the pYES2 vector (Invitrogen, USA) to generate the construct pY-MiPDAT. This recombinant vector and the empty plasmid pYES2, the negative control, were separately introduced into the mutant strain H1246 by electroporation (Bio-Rad, USA). Transformants were selected on SC-uracil agar plates and then inoculated in SC-uracil medium with 2% glucose on a shaker (220 rpm) at 30 °C. Following incubation for 24 h, the transformed line was collected by centrifugation and resuspended in SC-uracil medium with 2% galactose as an inducer for another 48-h incubation at 16 °C.

### BODIPY staining of yeast cells

BODIPY 505/515 (Life Technology, USA), a specific dye for the assay of neutral lipids, was used to stain yeast oil bodies as described previously^23^. The fluorescence image was captured by a confocal laser scanning microscope (Carl Zeiss, Germany) with a 488 nm excitation filter.

### Extraction of lipids from yeasts and TLC analysis

Total lipids were extracted from yeasts according to Blight and Dyer^41^. The sample was resolved in 30 μL of chloroform and pipetted by capillary onto TLC Silica Gel 60 F254 plates (Merck, Germany) and developed with hexane/diethyl ether/acetic acid (80:20:1, v/v/v). The plates were visualized by spraying with a solution containing 8% phosphoric acid (v/v) and 10% CuSO_4_·5H_2_O (w/v) and subsequent heating at 140 °C for 10 min.

### Feeding experiment for substrate preference and GC-MS analysis

To test whether MiPDAT had a substrate preference, LA, ALA, GLA, and ArA, the main FAs in *M*. *incisa*^17^, were selected in this feeding experiment. The transgenic yeast was incubated with 200 μM FAs and 0.01% (v/v) tyloxapol (Sigma-Aldrich, Germany) as a surfactant in the induction medium described above and harvested at an optical density of 3.5. Approximately 150 mg of lyophilized yeast cells were used for lipid extraction and TLC analysis as described above. The recovery of TAG-containing fractions and the preparation of FA methyl esters (FAMEs) of the recovered TAG were according to the previously described method^23^. Fifty micrograms of heptadecanoic acid (C17:0, Sigma-Aldrich, USA) was added as an internal standard.

GC-MS analysis was performed with an Agilent 7890-5975b system (Agilent Technologies, USA). The detailed GC-MS conditions are provided in the Supplemental Methods. The mole number of FAMEs was calculated with the peak area of the internal standard.

### Preparation of yeast crude extracts and *in vitro* MiPDAT activity assay

The crude extracts of transgenic yeast were prepared as described by Dahlqvist *et al*.^9^. The extracts were resuspended in the storage buffer (50 mM potassium phosphate, pH 7.2, 10% glycerol) to a protein concentration of 5 μg μL^−1^. The *in vitro* assay of MiPDAT activity was performed as described by Liu *et al*.^4^. The reaction mixture containing 250 μM lipid donors (PC or PE), 250 μM lipid acceptor (DAG), and the resuspended crude extracts (40 μg protein in total) with 50 mM potassium phosphate (pH 7.2) in a final volume of 200 μL. Reactions were incubated at 30 °C for 1 h and the lipids were extracted and detected as described above. The crude extracts alone and crude extracts with either lipid donors or acceptor were used as controls.

### Lipidomics analysis of *Myrmecia incisa*

Chlorophylls in the total extracted lipids described above were removed by solid phase extraction (SPE) with CNWBOND Carbon-GCB SPE columns (CNW, Germany). Reversed-phase analysis of lipids following lipid extraction was performed on a Dionex Ultimate 3000 High Performance Liquid Chromatography (HPLC) system (Thermo Fisher Scientific, USA) by injection of 4 μL of sample onto a Kinetex C18 column (100 × 2.1 mm, particle size 1.9 μm; Phenomenex, USA). The detailed LC-MS conditions are provided in the Supplemental Methods.

Raw LC-MS data for all samples were initially processed using the Thermo SIEVE 2.1 Qualitative Analysis Software (Thermo Fisher Scientific, USA). The data from each sample were then normalized to the total area, and all data with peak numbers [based on the retention time and mass-to-charge ratio (m/z)], sample names, and normalized peak intensities were imported into the software SIMCA-P+12.0 (Umetrics, Sweden). Multivariate analyses, such as PCA and OPLS-DA, were applied for the classification of lipid samples. The quality of the model OPLS-DA was evaluated by two parameters, R^2^Y(cum) and Q^2^(cum), according to Wiklund^42^. If the value of the latter was higher than 0.9, the model was considered excellent^42^.

### Identification of lipid metabolites

To select potential lipid biomarkers that contributed to the separation between the 0 d and 4 d groups grown under nitrogen starvation stress, three criteria, specifically, the high variable importance in the projection (VIP) and jack-knifed confidence interval (CIJF_JK_) excluding zero and an absolute value of p(corr) higher than 0.4, were used^29^,^30^. The lipid metabolites were identified using a commercial MS/MS database, Lipid Search (Thermo Fisher Scientific, USA). Changes in PC and PE were expressed as the relative abundance (%), which was the ratio of the peak intensity of the test group (4 d) to that of the control group (0 d) with the same dry weight, and the relative abundances (%) of the control was expressed as 100%^43^,^44^,^45^.

### *MiPDAT* transcription levels estimated by Q-RT-PCR

Quantitative RT-PCR was used to detect the *MiPDAT* transcription levels as described previously^39^. The cDNA was synthesized using the Reverse Transcribed Kit II (TaKaRa, China). The *Myrmecia incisa* β-actin gene (GenBank accession No. FJ548973) served as an internal standard. The primers used in the Q-RT-PCR were shown in Supplementary Table S1. The Q-RT-PCR amplification was performed in an ABI 7500 Real Time PCR system (Thermo Fisher Scientific, USA) with the SYBR^®^ RT-PCR Kit (TaKaRa, Japan). The relative gene transcription data from triplicate reactions performed for each incubation time were expressed as the means ± standard error using the 2^−ΔΔCT^ method^46^.

### Subcellular localization by agroinfiltration of tobacco leaves

To investigate the subcellular localization of MiPDAT, the complete ORF of *MiPDAT* was fused with *GFP* into a binary vector and then infiltrated into tobacco leaves using *Agrobacterium tumefaciens*. The PCR products for the *MiPDAT* ORF amplified with the primers pKpnF and pXbaR (Supplementary Table S1, containing *Kpn* I/*Xba* I digestion sites) were ligated into a modified pCAMBIA1300 binary vector containing triple GFP molecules (from Prof. Z.-N. Yang) in tandem (designated p1300-MiPDAT-GFP). This recombinant vector and an empty plasmid, p1300-GFP, as the negative control were separately introduced into *A*. *tumefaciens* GV3101 by electroporation (Bio-Rad, USA) and then infiltrated into the abaxial side of leaves of 4- to 5-week-old *Nicotiana benthamiana* plants^47^. After agroinfiltration, the transgenic line was placed in the dark for 48 h. The epidermis cells of the infiltrated tobacco leaves were observed under a confocal laser scanning microscope (Carl Zeiss, Germany). GFP fluorescence was monitored with a 500- to 550-nm band pass emission filter excited at 488 nm, and the autofluorescence of chlorophyll was examined with the same excitation filter as GFP, but with a 650 nm to 750 nm emission filter.

## Additional Information

**How to cite this article**: Liu, X.-Y. *et al*. Phospholipid: diacylglycerol acyltransferase contributes to the conversion of membrane lipids into triacylglycerol in *Myrmecia incisa* during the nitrogen starvation stress. *Sci. Rep*. **6**, 26610; doi: 10.1038/srep26610 (2016).

## Supplementary Material

Supplementary Information
