# Supplementary material for: Phospholipid: diacylglycerol acyltransferase contributes to the conversion of membrane lipids into triacylglycerol in Myrmecia incisa during the nitrogen starvation stress
Source: Sci Rep. 2016 May 24;6:26610. doi: 10.1038/srep26610 (PMC4877601; doi:10.1038/srep26610)
Supplement: Supplementary Information [file srep26610-s1.pdf]

Supplementary Information

Phospholipid: diacylglycerol acyltransferase contributes to the conversion of membrane lipids into triacylglycerol in *Myrmecia incisa* during the nitrogen starvation stress

Xiao-Yu Liu, Long-Ling Ouyang, Zhi-Gang Zhou*

College of Aqua-life Sciences and Technology, Shanghai Ocean University, Shanghai 201306, China

*Author for correspondence: e-mail zgzhou@shou.edu.cn. Tel: 0086-21-61900424, Fax: 0086-21-61900405

Supplementary Methods

Bioinformatics analysis

ORF Finder (http://www.ncbi.nlm.nih.gov/gorf/gorf.html) was used to predict the coding region of *MiPDAT*. The intron and extron of *MiPDAT* were analyzed using Spidey (http://www.ncbi.nlm.nih.gov/spidey/). Signal peptide site of MiPDAT was predicted by SignalP 4.1 Server (http://www.cbs.dtu.dk/services/SignalP/), while transit peptide site of this protein was predicted by TargetP 1.1 Server (http://www.cbs.dtu.dk/services/TargetP/) and ChloroP 1.1 Server (http://www.cbs.dtu.dk/services/ChloroP/). Prowler v. 1.2 (http://bioinf.scmb.uq.edu.au:8080/pprowler_webapp_1-2/) was used to predict subcellular localization. TMHMM Server v. 2.0 (http://www.cbs.dtu.dk/services/TMHMM/) was used to predict transmembrane domains. Functional analysis and classification of protein was predicted by InterPro (http://www.ebi.ac.uk/interpro/scan.html). Protein sequences of PDATs were retrieved from NCBI (http://www.ncbi.nlm.nih.gov/protein/?term=dgat), and phylogenetic inference was constructed using MEGA 6.0 program with the neighbor-joining (NJ) method[1]. Homologous sequences of PDAT from different organisms were aligned by ClustalX with default parameters[2].

GC-MS condition

The GC column was a fused silica capillary column coated with HP-88MS (60 m × 250 μm × 0.25 μm). The GC program was as follows: the initial oven temperature was held at 70 °C for 1 min, then heated to 210 °C at 10 °C min^{-1}, and finally heated to 235 °C at 5 °C min^{-1} and held for 15 min. The injection volume was 1 μL. Helium was the carrier gas at a flow rate of 0.6 mL min^{-1}. FAs were identified based on comparison of their mass spectra with those stored in the NIST 08MS libraries.

LC-MS condition

The flow rate of column effluent into the electrospray ion (ESI) source was 0.4 mL/min. To produce ions that could be readily fragmented, 10 mmol/L ammonium formate were added to the mobile phase as the electrolyte. For efficient separation of the total lipids, acetonitrile/water (3:2, v/v) was used as the mobile phase A_2 and acetonitrile with 0.1 formic acid/isopropanol (1:9, v/v) as the mobile phase B_2. The initial composition of the mobile phase B_2 was 30% and held for 2 min, and then increased to 100% in 18 min and held for 20 min, and finally returned to the initial 30% in 0.1 min and equilibrated for 5 min. In this experiment, the negative ion model was used to detect PLs on a Thermo Fisher Scientific Orbitrap Elite mass spectrometer as described by Nakanishi *et al.*[3]. The mass range was from 200 to 1,500. The heater temperature was set at 300 °C and capillary temperature was at 350 °C. The sheath gas flow rate was 45 arb, the aux gas flow rate was 15 arb, and the sweep gas flow rate was 1 arb. The spray voltage was set at 2.5 kV.

Supplementary References

1. Tamura, K. *et al*. MEGA6: Molecular evolutionary genetics analysis version 6.0. *Mol. Biol. Evol*. **30**, 2725-2729 (2013).
2. Thompson, J. D. *et al*. The CLUSTAL_X windows interface: flexible strategies for multiple sequence alignment aided by quality analysis tools. *Nucl. Acids Res*. **25**, 4876-4882 (1997).
3. Nakanishi, H., Ogiso, H. & Taguchi, R. Qualitative and quantitative analyses of phospholipids by LC-MS for lipidomics. In: *Lipidomics*, Vol. 1 (ed. Armstrong, D.) Ch. 15, 287-313 (Humana Press, 2009).

Supplementary Table S1

Table S1. Primer sequences used in this study.

Primes	Nucleotide sequences (5’-3’)
RACE for cDNA cloning	
GSP5-1	CAGCACTGCGTGTCCATGAGGAACTGCTGC
NGSP5-1	GACCACCGCAAAGCCCGTCCGGTTGCAG
GSP3-1	GCGCCTACCACTACCTGCACATGCAGGA
NGSP3-1	GGAAGATCAACACCGAGGTCTCGCATGAG
DNA cloning	
MiPDAT-OS	ATGGCTAAAGATGCTGCACAA
MiPDAT-OA	CTACAGGTCCGCTTCAAGGT
Quantitative real-time PCR	
MiPDAT-QS	TCTACCCAGTGGAAGAAGTTG
MiPDAT-QA	GTACAGGGATGGCTACTACAC
Miβ-actin-QS	CGTCCAGCTCCACGATTGAGAAGA
Miβ-actin-QA	ATGGAGTTGAAGGCGGTCTCGT
Heterologous expression	
pHindF	AAGCTTATGGCTAAAGATGCTGCACAA
pBamR	GGATCCCTACAGGTCCGCTTCAAGGT
pKpnF	GGATCCATGGCTAAAGATGCTGCACAA
pXbaR	TCTAGACTACAGGTCCGCTTCAAGGT

Note: the underlined letters indicate the position of restriction enzyme sites.

Supplementary Figure S1

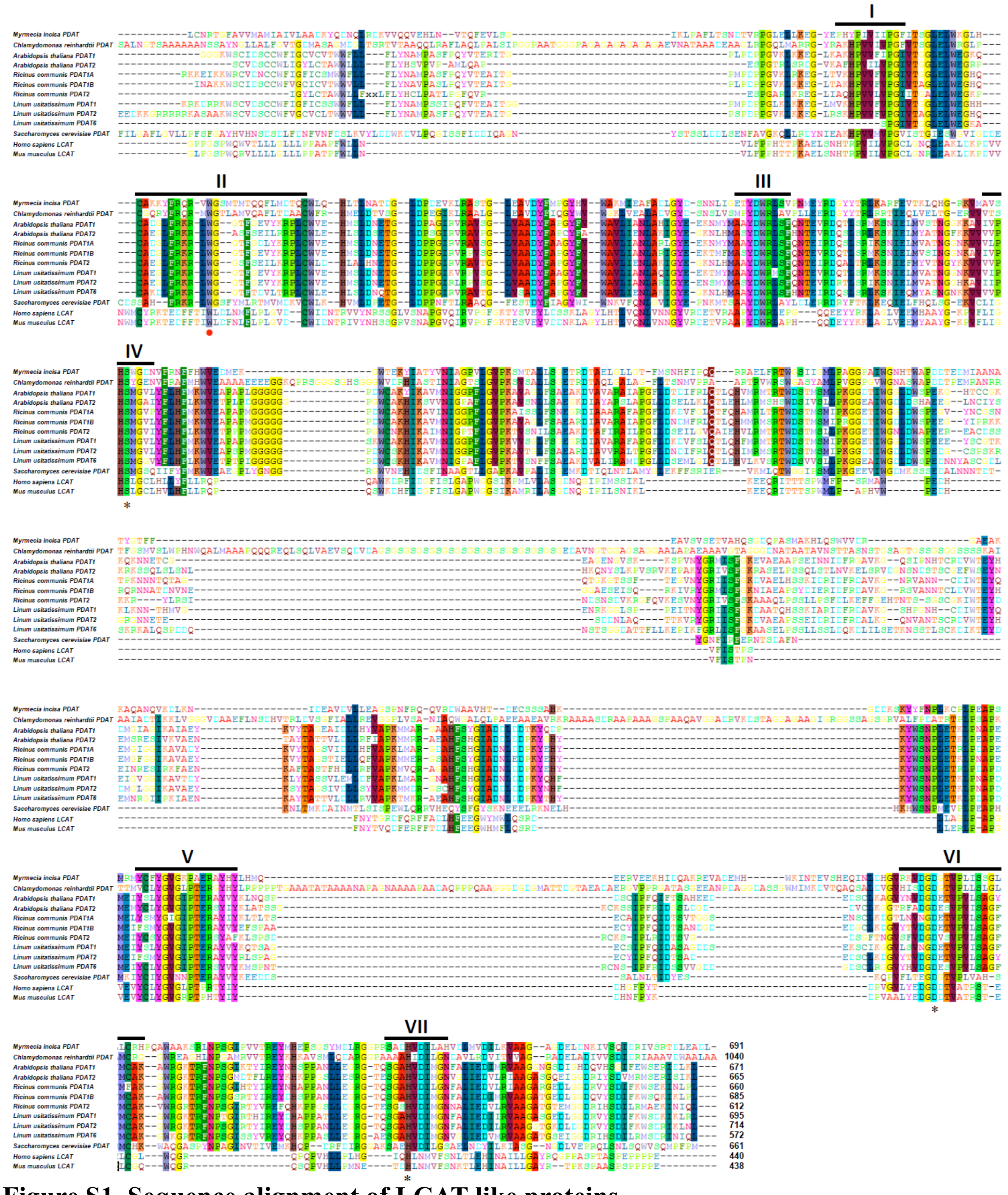

Figure S1. Sequence alignment of LCAT-like proteins.

The seven most characteristically conserved domains are shown. The accession numbers of the protein sequences in this figure are the same as those in Figure S2. A Trp 285 residue in the lipase lid domain involved in binding the cleaved fatty acid in the active site of the enzyme is marked by a dot. The catalytic triad (Ser-Asp-His) is marked by an asterisk.

Supplementary Figure S2

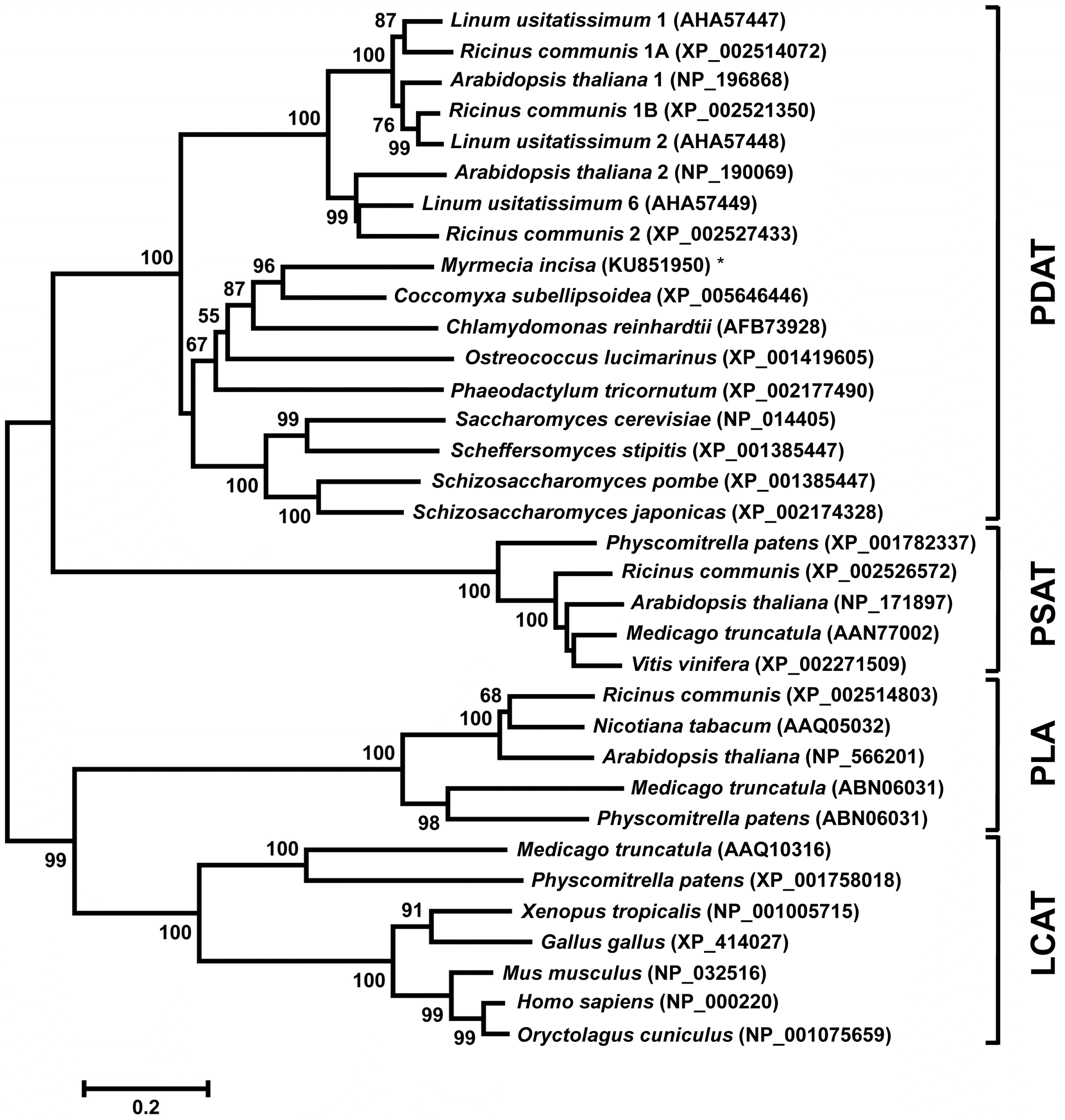

Figure S2. Phylogenetic tree of the LCAT-Like proteins.
All of the accession numbers are presented in the phylogenetic tree. Branch lengths are proportional to the number of substitutions per site (see the scale bar). The numbers at the nodes indicate the neighbour-joining bootstrap proportion (BP) values (only values ≥50% are shown). The MiPDAT is marked by an asterisk.

Supplementary Figure S3

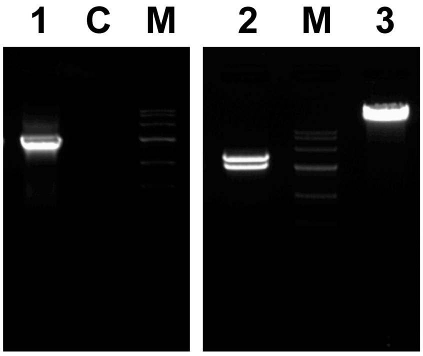

Figure S3. Agarose gel electrophoretogram of products during the construction of pY-MiPDAT.
Lane M: DNA Marker IV; Lane 1: products of colony PCR of recombinant plasmid pY-MiPDAT; Lane 2: double digested products of recombinant plasmid pMD19T-MiPDAT with *Hin*d III/*Bam*H I; Lane 3: double digested products of plasmid pYES2 with *Hin*d III/*Bam*H I; Lane C: water as the template as a negative control.

Supplementary Figure S4

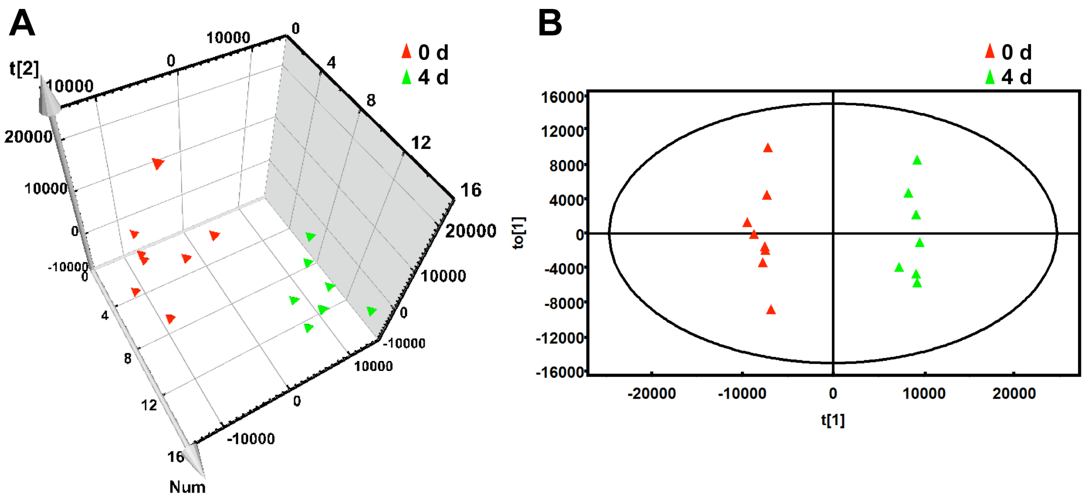

Figure S4. Multivariate data analysis of lipid data from 0 d (culture at the onset of nitrogen starvation) and 4 d (nitrogen starvation cultivation for 4 days).
(A) PCA score plot of data from 0 d and 4 d for the first two components. (B) OPLS-DA score plot of data from 0 d and 4 d.

Supplementary Figure S5

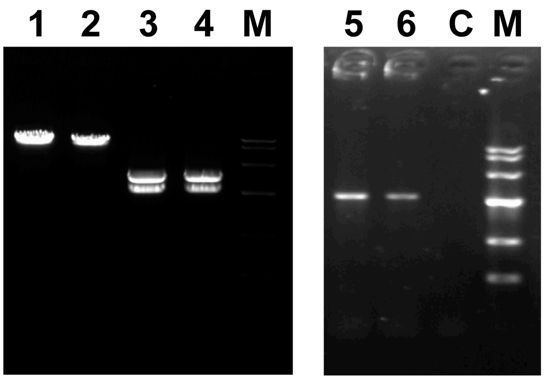

Figure S5. Agarose gel electrophoretogram of products during the construction of p1300-MiPDAT-GFP.
Lane M: DNA Marker IV; Lanes 1 and 2: products of colony PCR of recombinant plasmid p1300-MiPDAT-GFP; Lanes 3 and 4: double digested products of recombinant plasmid pMD19T-MiPDAT with *Kpn* I/*Xba* I; Lanes 5 and 6: double digested products of plasmid p1300 -GFP with *Kpn* I/*Xba* I; Lane C: water as the template as a negative control.

Supplementary Data 1

4 unigenes coding for ORP protein

>Contig548 (768 bp)
ref|XP_001699422.1| oxysterol binding protein [*Chlamydomonas reinhardtii*],
identities=59%, E value=$4e^{-94}$
CCTGTGAGGACCCACACGAGCGGCTGGCGTGGGTGGTCGGCTTCTTCATCGGC
GTGTTTGGCAGCCTGGAACGCACCTGGAAGCCGTTTAACCCCATCCTGGGCGA
GACCTTTGAGCTGGACCTCGAGAACGGCACCCGGTTCTTGGCAGAGCAGGTGA
GCCATCACCCGCCCATCGGTGCCGCGCATGCCGAGAACACAAACTGGACGTAC
GACATCGTGTCCGCACCGTCCACGAAGTTTCTCGGCAACAGCGTGGAGATCTAC
CCCGTAGGCCGCTCGCGCCTCACGCTGCGGCGCACAGGCGAGGAGTTCACACT
GACCCCGCCCAACACCAAGGCCAACAACGTGGTCATTGGCCGCACCTGGATCG
ACACGGCCGGCGACTTCACGCTGCTCAATTGCACCTCCGGCGCGAAGTGCGCA
CTCACCTTCACCCCGTGCGGCTGGTTCGGCAGCGGACGCTACGAGGTGTCAGG
GCACATCCTGGATGCGGACGGCAAAAAGGTGCTCGCCCTCAACGGCAAGTGGA
ACTCCTACCTGGAGATGGTCAAGTGCGATGAAGCAGGGGAGCCGCTACCTGAC
GCCCCTACCATCCGGCTGTGGGAGTGCAAGCCCAAGCCTGAGAACGACAAGTA
CGGGTTCACGTTTTCGCGCGTATGCTCAACAGCTGCAAGGGCATCAACCCTAT
GCCCTCTGACTCGCGCCGCCGCCCCGACCGCGCCGCCTTGGAGGCGGGCGACA
ACCTGACGGCTGGCGCTGAGAAGT

>Contig8824 (779 bp)
gb|EFA85771.1| oxysterol binding family protein [*Polysphondylium pallidum* PN500],
identities=37%, E value=$2e^{-56}$
CTTGCCGGAAGCGCGAGTCGCTCTCAAGTGGCGCTGTGATGGGCGCCGGCGAG
CGCACAGGCGAGGTGTGCACATCCCACAGCCGCCGCACCTTGCCGCCGCGCAC
GCCGCGTTCCCACTCCACATGCGTCAGCCAGCTGCCCGACACGCTGTCGAGGA
CCTGGCCGCGCTGCACCAAGCTGCCACGCACAGCGTCCTGCTGCGCGCGCTGG
GACTTTTTGGAGCGCCAGAGCGCCGAGATGAAGCTGGCCGCAGGCTGCAGGTC
GACCCGCACCACGCAGCTGAGGTTGTTCAGGTCGTCCTGGAAGTGGATTTCAC
CGGAGTACTTCAGCACACGCTCGCCCCACATGATGCCTCTCAAATGCAGGGGTG
GCAACTCCCACGTGACTGTTGCGCCGTCGCTGGCAAACCAGCAAGCCTGCTTG
CCACTTTGCTGGCCCTTGATGGAGTTGCCCCGCGCCGCCGCGACCCAGTTGCCG
TTGCCATAGAAGCGGAACGTGTTGTGCTTGTCCCTAACATCCCAACTGCTGATT
GGCGGGTGGTGCGAGATCTGCTCTGCTGCCACTGTCACACCCGAGCTGTACTCT
GACTGGTGCGTCTCGCCCAAAATGGGGTTGAACGGCTTCAGAGTGGAGGTAGC
TCTCCTGAGGCCGCCAATGACAAAGGCTACCAGGTACTTCATCCGGTCTGCTGG
GTCTGCTGTGGCTGCTGCAATCTCAAGCAGGTCCAGATAGGCCCAGTTGTCTGT
GATGCGCTGCAGGAAGCTGCGTGTCGAGCGT

>Contig18581 (593 bp)
ref|XP_001346953.1| oxysterol binding protein [*Paramecium tetraurelia* strain d4-2],
identities=41%, E value=$5e^{-24}$
ACGCTCGACACTCCCCAGTGTCCTTAGACTCCGTCTTGGCCATGGACTTTGACG
TGACTGTCATGCTTGCAAACTTGGAGCCCAGCCTGGTGAGGCTAGAGCTCCGG
GAGGGGGCCTGCGCACTCGCTGTATCCAGGCGCACAATTTGGCCGCTGAAGGC
GTCGGGCCGCTGCAGAAGCCAGTGGCTCTTGCCCGGGACTTTGCCAAACGTGA
TCTGGCACTGCAGGTGGTGCGCTGCGTCCTGCACTGTGAACGCTCCAGTCATCT

CGCCATGAGGCTGCTCGCTGTACAGGATACCTTTTAGCAGGTACGACGGAAAGT
CCATGTGCAGCTTGACACCGTCTGCAAACGTAATGCTGCGCGATCCACGGGTGA
CAGTCTTGATCGCGTTGGCCTTGTAGTGCACCTCTGGGCGGGAAAGGCCTTCGA
GCGTCCATCCTGAGCCCAACAGTTGGTAGGCGGAGACTGGAGGGTGATGTGAG
ATCTGCTCCATGTACACACGCGTCCCGGATGCAGTGCCTGCCTGAAAGGTCTCC
CCAAGTATGGGGTTGAAAGGCTTCATCCACGAGGCAAACGCAAACTGCTGACC
AGCT

>fu-GY9GJ1Z02H04PU (239 bp)
ref|NP_200750.1| oxysterol binding protein-related protein 3C [*Arabidopsis thaliana*], identities=67%, E value=1e^{-12}
GCCAGCCGCTCGTGTGGGTCCTCACAGGCGGACGCCTTGTCGAGCGCCTCATT
GTACTCCATGATCTGCGCCATGGTCTGCAGGATGGTGAACGGCTCCATGATCCA
CACGGGCAGCGTGACAAGCGACGTGATGTCCATGCCGATGTAGTCGACGGTAG
TTGGNGAACGCCGCCTGCCGCTGCGCCTCGTCAATACCTCCATCGCCCCTTGGT
GGAGTAGTCGCATGACTCTGCCTGG

Supplementary Data 2

2 unigenes coding for Sec14 protein

>Contig19489 (2471 bp)
ref|NP_195382.1| sec14p-like phosphatidylinositol transfer protein [*Arabidopsis thaliana*], identities=44%, E value=1e^{-58}
GGTTATTGCAGAGATTGTCAAAAGAGAGGTGTGCTCGTGGTGTCATATTTCTGG
ACAATGTGGTCCCATCTGCGCATCGTACATTTTCGTCACAAGAAAACTCGGGAC
GTGTGACACATGAAGGGAGCCTGCCATGCAAAAGGAAGCCTGTTTGTGGTAAC
AGCAAACCTGCGACTTGGGAGGGCACCGAGCCACACTCAACCACCAGATCGAC
GAGACATAAGCACAAAGGTGAGTCAGAAACCTAACCATGTCTATGTCAGGGTCA
TGCTGTATTTTTATCCAGTCGTCAGTATGGCAAGCATCACTCATCACCACGAGGA
CGCTACCTACAAGTCGTATCAAGCATCTAGTAAGCGCAAGATTGGCAACTTCAC
CTCTAGCATGCTGCAACCGGGCCAGCTCACCGCAGCCTATTCCCGATGGATATAC
TGAGCTTGCAACACCAGGCGCCTGTGCGGGTTTGGCGACAGCTAGGATGGGGC
AAGGTCACTTAGCCCCCCTGCTAAGTTTGTGTCAAAGCGCACATAAGGTGTCTT
TGGAACAGCTGTGCCAAAGAACACAGCGCTACCCATAGCTATGGTCACTAGTAC
ATACACAGCCTAGGTAGCGACAGAGCGCCAACCCAAGCCAACCGGATCGCACT
TCCCTGCAAAACTGCTCACTTGACAGGCTGGGAGGCCTGATCCCAACAAGGGG
CCCTCAGCATATGTGGCAGGTGTCTGCAAGTCAGGACTGCCACCTCAAGTAACC
ATCCATGCATCACTCGCCATGTTCCACGCTGTACCCATACGTCCACCTCAAAGAC
TGCCAGCGAGCACACTAAAGGGTTACACGCAGGGGTTGCCACAAAACGCGCA
GCACGGTGCTACATATGCTACGATCCACGCTCTTAGGATGGGATTAATGCTAGTT
GGATTGCGGCTGCCACCCCAGGGGGCCTGCAAAGCCGGAAGGGCGCACAACA
CCACGAAGGGTCCCCTACATCGTGTGATGACATCTCATCGCATGACAGTAGCAC
CCCCGCGTTAATTGACAAGCGCACAACACCACGAAGGGTGCCCTACATTGTGTG
ATGACATCTCAACGCATGACGGTAGCACCCCCTCGATAACTTACAAGCTGCCCT
GCAATTAGGGCCTAAAAGGCAGCGCCAACTGCCGCATGGTGCGGGCTGCTCTC
CTGCAGGGTGTGCCGCTGCATGCCATTCGTGATGCCGTTGCTGCGGTCGTCCAT
CTCCAAGCGTCCCTCCTTGATAGGGCCATGCGTGGTCACTACCTGCTGCTGCAG
CCCCTCTGCGCCAGAGGTGCCGTTGTACGCGGCTGTGCTGCTAGCAGCGCTCTC
CACACCCTTGCCAGCCTGGACAGCCCCGCCGTTCGACAGTGGGTGTGCGTAGG
TGTCCACAGCAACCTCCGCCTGCCTGCTCGGCTCTGCAGCCTCTACCACGCTGC
CGCGGTCGGCGGGTTCGGCGTACGCCATGCCCTCGCCAGACGCTGCGACCAGT
TTGTTGACCGCACCACCGGCTGCCGGCGGCTCGAGCGCAGCGATCTGCTTGTC
GCGCAGCTCATCCTCCTGTATCATGCTGCGGCTGTAGGCGTCCAAGTCGAACTG
CCATGTGGACGTGCCCCCAAAGCACTCCTCCAGCTCCGCCATGTCAAACTTCTG
AGCCATGAGCTCGGCGCCAGCGGCCGTCTTGTCCACCACGCAGACCTTCTTGA
GCGTGACGGGGTCGATGAAGGGGCGCACCGCCTTCCACATGAGGTTGAAGAGG
CGGGGCGGGTGGTAGTAGACCGACAAGCCCAACCGCTCCGGGTAGTGGTTCTG
CAGGATGGACGCGGTCTGCAGCGCAACCTTGAGCGGCGGCGCGGTCGCCATCG
TGTAGCCCTCAAAATCGATCAGCCAGGTCATCTTACCCACGCCTTCTCGATCAGC
GGCTTGTGAGGATGCCTCTAGGTGGTAGACTAGCATGCGGATGCCCGCCACGTA
GTTGTCAGAGTTTTGACACCTGGGCCGCATGACCAGCACGACACGCCCGTCAC
GGTCGCGCGCCTGCATGCGGAACTGCTTGCCGGTGGCAGCCTCCGCCTTGACCT
CCGCCCACGTAATCTCGTGTGGCTTGTACGTCAGCCGCCACTCTATGGTGGCCTT
CAGCATCTTCGTTGCTTTGGCAAGGTCCCACTGCCGTGCTTTGAGGTACCGGAC
GAGGGTCATCTGTGTGCACCACCATCGCAGCGCGGCCGAGCTCTCCAGACTGG
TGGCAACGGCGGCTCTCAGGTCGGCCACACTCTGCTGCTGCTGAGCAGTGAGT

GGCTCTGCCTCCGTCATGCCTTCAAGCTGTCGCGGTGACGCAAGCGCACGGAA
GCGAACGCAAACAGCCGGTGCGGAACTCCTCGACCGTTCCGCAAGCCTCAGCT
CCGCAAGGAGTGTTGGGGACTTTTTGGTAACCTCCCGTAATCAGGTGTGTCGAG
CGT

>fu-G624HKO04IVGQ8 (271 bp)
ref|XP_002874544.1| SEC14 cytosolic factor family protein [*Arabidopsis thaliana*], identities=38%, E value=$3e^{-12}$
ACGCTCGACACTTCCAAGTCAAAAGCACCAGTCTGAGGACCCCCCGAGGCACT
TCTCCATGCAGCCCATGTCAAACAACTGCGCCATGAGCTGGCAGCCGGCTTTGC
TGTTGGCCGCAAAGAGCACCTTCTTGATGGTCACGGCGTCCAGAAATGGCCTCA
CAGCCCGCCACATGGCGTTGAAGAACCAGGGGGCATGGAAGCAGACAGCAAA
CCCTAGGCGTTCGGGGTAGTGGTCGGTGAACAGATGGATGGTCTGCATCGCGAC
GCTC

Supplementary Data 3

2 contigs coding for coatomer proteins

>Contig1247 (897 bp)
ref|XP_002501894.1| coatomer protein complex [*Micromonas*. RCC299], identities=74%, E value=4e^{-111}
CCATCTCCCAGCCGCCCTCTTCATCGTCGCCCTCTGCACCGTCTGCGTCCACCA
GCACCCCGTCGTCCTCGCCAGCCCGCGGCGAACCGCCCCCGAGGTCGAGCCCA
TCGTCGCCCCAGGCAGCGCCCGCCACGTCCAGGTCCTCCTCTTGGAGGCCCCC
CAGGCCCGCATCCGCCTCTTTGGCGGCCAGGTTCTCAAAGAAGCCCTTGCTGAC
GGTAAGCAGCGGCCAGTTGTCCTCCTTGAGGATGGGCGTCGGCGGCAGCAGCA
GGCGGGCATCGGGGTCCAGCTGCGGAAGCTCGGGTAGTGACTCGGCCAGCCGT
GCGGTGTCCTCCTCCAGCCCATGGGTGGCTGCCGTCACATACGCCAGTGCAGTT
TGGCCTGTCTCCTCCAGGATGCGCACGCGCTCGCGCACATCCCCCAGGTACAGC
GCATTGTGGAAGCGGCCCATCACGTCGTTCCGCATCTCGGCGATCTTGAGCATC
TTGGCCAGCTTCTCCAGGTTGCCCGTGATGAGGTACAGGAAGGACAGCCGCTC
GAAGTTCTTCGTCTTCTGGTAGCTGAACTCCACAATCTGGTGGTTGCCCTGGCG
CAGTGCCTCCACGCCTAGCCGATGCCAGGTGTCCCGGTCGTCCAGGTCCTGCGC
GGACTGCAGCGCGACCTCGATGTTGCCGCACTCGATGGCTAGGTTAAAGCGCGT
GCGCTCGTCCTTGACGAAGTGCAGGGCGACCTCCGGGAAGCCCTTGGCCTGCA
GGTAGGCGATGATGGACTGGCCGCACAGCGCGCTGCCGCGGATCATGGCCAAG
ACCTGGTCAAACTGGCGGTTCATGAGCGCCAGCTTGAACATGAACTCCGTCGTG
TCACCTGGATCTGGCGTTCTTGCCGTCGCGGTCCAGGCAG

> Contig12219 (692 bp)
ref|XP_002507482.1| coatomer protein gamma-subunit [*Micromonas*. RCC299], identities=78%, E value=4e^{-67}
ACGCTCGACACCCGTGCAACGTGCCTGGCAAGTGTCACCAGGAGTACCATCGT
AAGCTCTGGGGTTCGCTAGACTGGTTTACATTACGTCCAGAGTGGCAGCAACTT
CAATCCAACCGACTGTGGGCTTTTAGTTGTGGTAGCTTCGGAAGGCAGGGTCCT
CATCCCGAAGGGGCCACACCCCTTTGAGCAGCGGCGATGGCGTCAACCGCCCA
GGACCCCTCTGCACCCCTGCGAGGGCCCGAGAAGAAGGATGAGGACAGAGAT
GAAGACACGGAGTACTCGCCCTTCTATGGCATCGAGAAGGGTGCCGTTCTGCA
AGAGGCTCGGATATTCAACGACTCACACATAGATCCCCGCAAGTGCCAGCAGGT
CATCACCAAGCTCCTCTACTTGCTATGCCAAGGAGAGACGTTCAGCCAGAAAG
AGGCCACCGAGGTGTTTTTCTCGGTGACCAAGCTATTCCAGAACAAGGACGCC
AACCTGCGCCGGATGGTGTACCTGGTGGTCAAGGACGTCTGTCCTGCATCCGAT
GAGGTCATCATGATCACCAGCTCGCTGATGAAGGACATGAACAGCAAGACGGA
CCTGTACCGGTCCAATGCCATCCGCGTGCTGTGCAGCATCACCGACGCGCAGCT
GCTGGGTCAAATAGAGCGCTACCTGAAGCAGGCTGTCGTGGACAAGAGTCCG
